# Analytic Solutions and Entropy Production of the Double-Diffusive Equation System

**DOI:** 10.3390/e27090946

**Published:** 2025-09-10

**Authors:** Imre Ferenc Barna, László Mátyás

**Affiliations:** 1 Hungarian Research Network, Wigner Research Centre for Physics, Konkoly-Thege Miklós út 29-33, 1121 Budapest, Hungary; 2 Department of Bioengineering, Faculty of Economics, Socio-Human Sciences and Engineering, Sapientia Hungarian University of Transylvania, Libertăţii sq. 1, 530104 Miercurea Ciuc, Romania

**Keywords:** self-similar method, double-diffusion, salty fingers, fluid flow, heat conduction, 02.30.Jr, 66.10.Cb, 47.10.g, 35C06, 35G20

## Abstract

We investigate the partial differential equation system which describes the double-diffusion convection phenomena with the reduction formalism. Double-diffusion refers to when two scalar quantities with different diffusivity, such as heat and solute concentration, contribute to density gradients within a fluid under the influence of gravity. The time-dependent self-similar trial function is applied and analytic results are presented for the dynamical variables and analyzed in detail. Additionally, the entropy production was derived as well. In the second part of the study we investigate the role of an additional heat source.

## 1. Introduction

There is no question that transport processes have extreme importance both for scientific and engineering applications. The simplest ones are heat conduction in solids and the regular diffusion of particles. The existing literature of diffusion (or of heat conduction) is immense; therefore, we mention some recent basic monographs [1,2,3,4] and additionally some non-conventional ones [5,6] as well. The development in numerical analysis of diffusion equations made remarkable steps in recent years as well [7,8]. On the other side, the mathematical generalization of the diffusion equation including the p-Laplacian was also investigated [9,10].

Coupling additional transport mechanisms (like fluid flow) to regular diffusion or heat conduction drastically opens the horizon of interesting phenomena and non-linear effects. Good examples are simplified heated flow systems like heated boundary layers [11] or the Rayleigh-Bènard convection [12]. These are diffusive convection systems where heat is transported together with particle flow, which is convection instead of conduction. Such processes are extremely important in the science of meteorology [13], oceanography [14] or in climate change studies [15].

More than half a century ago, E.N. Lorenz investigated the Rayleigh-Bènard convection [16] with a truncated Fourier series as a trial function and he pioneered the way to a new discipline called chaos theory. On the other hand, if we investigate such diffusive convection systems with the self-similar Ansatz, we can relatively easily derive analytic solutions which can predict the asymptotic temporal or spatial behavior of such physical phenomena. The self-similar Ansatz is the natural trial function of the regular diffusion equation [17] because the fundamental or Gaussian solution can be derived in a few lines and astonishingly new kind of solutions can be easily obtained as well. This strong performance of this function gives us a strong hint that additional disperse dynamical systems can be successfully analyzed, giving insight into the global properties of their solutions. In recent years we successfully investigated such systems like the Rayleigh-Bènard convection [18] or heated boundary layers [19] presenting physically relevant analytic solutions in connection to different special functions like the Kummer’s M and Kummer’s U functions.

We can continue on this path defining more complex (or rather more compound) systems and investigate how they behave. First we analyzed systems where some diffusion equations were coupled in various ways [20].

In the next study we consider the double-diffusive convection which is a fundamental fluid dynamics phenomenon that arises when two scalar quantities with different diffusivity, such as heat and solute concentration, contribute to density gradients within a fluid under the influence of gravity. The interplay between thermal and compositional buoyancy forces gives rise to a rich variety of flow patterns and instabilities, which are often more complex than those observed in single-component convection. Understanding the mathematical structure and behavior of the double-diffusive convection equation system is therefore essential for both theoretical and applied sciences. Therefore a detailed linear stability analysis of double-diffusive convection was performed, laying the groundwork for understanding the onset of instabilities [21]. The low Prandtl number flow behavior which is relevant to astrophysical and geophysical applications was exhaustively studied as well [22]. The sub-microscale dynamics of double-diffusive convection was investigated recently by Radko [23]. The presence of certain particles in the fluid system may influence the heat diffusion [24]. Additional effects in homogeneous and heterogeneous porous media are also the subject of the current study [25]. The nature of diffusion may also depend on microscopic aspects of dynamics [26]. The double-diffusion phenomena is also associated with an irreversible entropy production, which expresses the dissipative nature of dynamics [27].

The literature of this field is remarkably extensive; without completeness, we only mention some relevant studies and monographs [28,29,30,31]. The double-diffusion process is an important process in oceanography in general [32], in geophysics, for understanding the phenomena in magma chambers [33], in astrophysics [22,34,35], or double-diffusive magnetic layering [36]. In hydrology it is meant to describe sediment laden rivers in lakes and the ocean [37,38], in metallurgy [39], or finally even in various engineering applications [40,41]. The formation of salt deposits, under salt density gradient and the presence of solar radiation, have been studied in [42], becoming a double phase problem at the point of crystallization [43]. The salinity gradient also typically implies a manifestation of bacterial diversity in lakes. Different bacteria may be present in lakes, depending on depth and salinity concentration [44]. The combined effects of salt diffusion, in the food industry, have also been studied [45].

After the very first analysis of ‘an oceanographic curiosity: the perpetual salt fountain’ by Henry Stommel and co-workers in 1956 [46], Stern in 1960 first described the double-diffusive convection and introduced the concept of salt fingers and their role in oceanographic processes [47].

One can see that one of the most studied double-diffusion systems is salty fingers. The question of the limits on growing finite–length salt fingers was analyzed and a Richardson number constraint was found by [48]. Planform selection in salt fingers was also studied [49].

Additionally—again without completeness—we mention some references for interested readers [50,51,52].

This publication aims to contribute to the ongoing exploration of the mathematics of double-diffusive convection by presenting new analytical results that shed light on the system’s behavior across different parameter regimes. By elucidating the underlying mechanisms and mathematical structure of this complex phenomenon, we hope to advance both the theoretical understanding and practical control of double-diffusive systems in nature and industry.

## 2. Theory and Results

### 2.1. Double-Diffusion System Without Extra Source Terms

**Figure 1:**
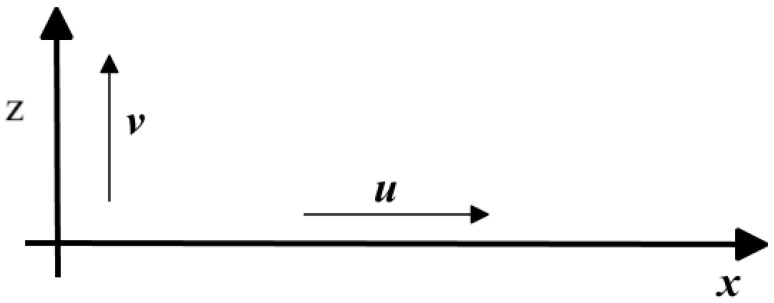
Defining the directions and the velocity components of the investigated system.

The conservation equations for mass, vertical momentum, heat and salinity equations (under Boussinesq’s approximation) which describes double-diffusive salt fingers can be formulated in general vector form [50]. We want to perform direct calculations. Correspondingly we start with the following system of differential equations:

(1)ux+vz=0,(2)vt+uvx+vvz−ν(vxx+vzz)+G(βSzz−αTzz)=0,(3)Tt+uTx+vTz−kT(Txx+Tzz)=0,(4)St+uSx+vSz−kS(Sxx+Szz)=0,

applying the standard notation; therefore, u,v,T and *S* denote the dynamical variables of the horizontal and vertical speed components, the temperature and the salinity. The subscripts refer to the partial derivatives in respect to the temporal and spatial variables. The physical parameters $\nu ,G,\alpha ,\beta ,k_{T}$ and $k_{S}$ are the kinematic viscosity, the gravitational acceleration, the coefficient of thermal expansion, the haline concentration coefficient at constant pressure and temperature, the molecular diffusivity of heat and the molecular diffusivity of salt [53]. We suppose that all the above mentioned coefficients are constant for the system studied. There are also cases where the diffusion or heat diffusion coefficient may depend on the parameters of the problem [54,55]. (The complete analysis or the realistic equation-of-state for sea water is a relatively complicated problem, having a large literature. Fortunately these aspects are irrelevant for our forthcoming analysis.) We consider Figure 1 to fix our system’s geometrical relations.

All five physical parameters should have positive real values. For better transparency we use subscripts for the corresponding partial derivatives. The horizontal and the vertical space variables are denoted with *x* and *z*, respectively.

In the next step, we apply the generalization of the self-similar Ansatz [56] for two Cartesian space dimensional dependent dynamical variables in the form of the following:

(5) $$\begin{array}{ccc} u(x,z,t) & =t^{-\mu }f\left(\eta \right),\;v\left(x,z,t\right) & =t^{-\gamma }g\left(\eta \right), \end{array}$$

(6) $$\begin{array}{ccc} T(x,z,t) & =t^{-\delta }h\left(\eta \right),\;S\left(x,z,t\right) & =t^{-\epsilon }i\left(\eta \right), \end{array}$$

where $\eta =\frac{x+z}{t^{\omega }}$ is the reduced variable. In the next step, we demand the existence of the corresponding first and second derivatives of the shape functions with adequate smoothness. For completeness, we mention that the very first use of $\eta =x/\sqrt{(}t)$ variable transformation was applied by Ludwig Boltzmann more than a century ago [57]. (We usually use the first two Greek letters α and β for the self-similar exponents as well but now these are fixed to physical parameters). The exponent ω is responsible for the spreading of the dynamical variable in time, and all the other four exponents describe the decay or increment of the variable in time. In most cases positive exponents mean spreading and decaying solutions in time, which meets our physical intuitions. Existing self-similar symmetry also means that the investigated system has no additional characteristic relaxation time or characteristic length. It is also true that self-similar solutions are defined on an infinite horizon and there is no need to introduce dimensionless variables like in the work of [50]. For an infinite horizon it is not possible to define reasonable Reynolds or Rayleigh numbers. It is worth mentioning that this self-similar Ansatz has a wide applicability in physics and it was successfully applied to explain the quickly expanding Universe in a recent article which is attracting remarkable interest [58].

After the usual steps of algebraic manipulations we arrive to the ordinary differential equation (ODE) system of the following:

(7)f′+g′=0,(8)−g2−ηg′2+(f+g)g′−2νg″+G(βi″−αh″)=0,(9)−h2−ηh′2+(f+g)h′−2kTh″=0,(10)−i2−ηi′2+(f+g)i′−2kSi″=0,

where prime means derivation with respect to η. Additionally we get some constraints among the self-similar exponents:

(11) $$\gamma =\delta =\epsilon =\omega =\mu =\frac{1}{2}.\;$$

Note that now all exponents got the same fixed numerical value, which means that the mathematics of the solution is quite restricted. If some exponents remain free then the ODE system and the final solutions contain them as free parameters too. For the regular diffusion equation, if both exponents are fixed to one half we automatically get fundamental Gaussian and error function solutions. It is worth mentioning here that the Rayleigh-Bènard convection model [18] (which in a sense is a far analog) of this system has slightly different self-similar exponents, resulting in a much richer mathematical structure. Now, the original physical parameters of the starting dynamical system remain free. As usual the ordinary differential equation of the shape function of the continuity equation Equation (7) can be integrated, giving us the following: f+g=c where *c* is our first free real integral constant which is proportional to the velocity of the flow. These conditions help us to decouple the heat and the salinity equations from the momentum and the continuity equation. We get even more; the heat conduction and the salinity equations become linear, not depending on the products of dynamical variables. Both become independent in the form of

(12) $$\begin{array}{cc} & -\frac{h}{2}-\frac{\eta h^{\prime }}{2}+ch^{\prime }-2k_{T}h^{\prime \prime }=0, \end{array}$$

(13) $$\begin{array}{cc} & -\frac{i}{2}-\frac{\eta i^{\prime }}{2}+ci^{\prime }-2k_{S}i^{\prime \prime }=0. \end{array}$$

**Figure 2:**
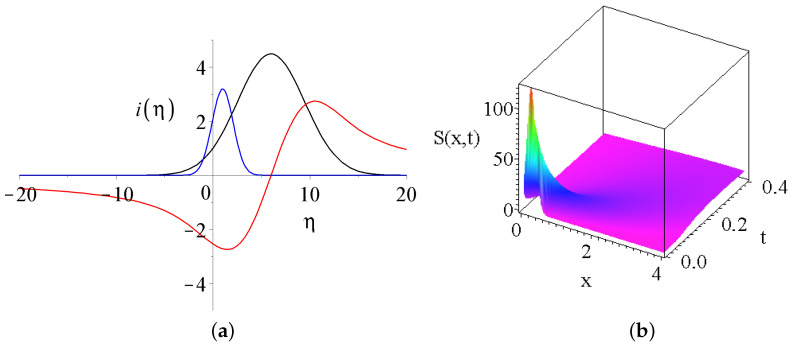
(**a**) The shape functions of the salinity equation Equation (15). The black and blue curves are for the real part of the solution $c_{3}=0$ for the numerical parameters sets of (1,3,3) and of (2.1,0.5,0.3) for the parameters of $\left(c_{4},c,k_{S}\right)$. The third red curve shows the imaginary part $c_{4}=0$ for the parameters of (1,3,3). (**b**) This shows the projection of the real part of salinity distribution S(x,z,t) for z=0, with the parameters of (1,3,3), respectively.

The solutions can be easily obtained by integrating the ODEs, giving us the following:

(14)h(η)=e−η2kT(η4−c)·c1erf14−2kTη+c−2kT+c2,(15)i(η)=e−η2kS(η4−c)·c3erf14−2kSη+c−2kS+c4,

where erf refers to the Gaussian error function, now with an imaginary argument, and the real integration constants are notated with $c_{i},\left\{i=1\ldots 4\right\}$. For more information about the properties of the error function, consult the handbook of [59]. Figure 2a presents the shape functions of Equation (15) for some parameter sets. The numerical value of *c*, which is basically the velocity, enhances the maximum of the peak and makes a shift to the right of the peak. The $k_{S}$ molecular diffusivity of salt is responsible for the full-width at half maximum (FWHM) of the peak. Figure 2b shows the projection of the S(x,z,t) salinity distribution (z=0). This is very similar to the usual Gaussian solution of diffusion.

Now, if the second derivatives of Equations (14) and (15) are derived and replaced into Equation (16) then the momentum equation of the double-diffusive convection problem can be solved. Note that the possible integration of the continuity equation may lead to a linear momentum equation, which will also mean that in the next step, the linear combination of solutions (superposition) will also give us further solutions. Our experience shows that fully analytic solutions can be evaluated only for $c_{1},\;c_{3}\neq 0\;\&\;c_{2},\;c_{4}=0$ in the reverse condition $c_{1},\;c_{3}=0\;\&\;c_{2},\;c_{4}\neq 0$. The solution contains an additional integration which should be evaluated numerically when all parameters have a given numerical value. No fully analytic solutions exist for the most general case where all integral constants are not zero. We analyze the real solutions only. Therefore the ODE for the velocity shape function contains just the second derivatives of the exponential function and reads as follows:

(16) $$-\frac{g}{2}-\frac{\eta g^{\prime }}{2}+cg^{\prime }-2\nu g^{\prime \prime }+G{\left(\beta c_{4}e^{-\frac{\eta }{2k_{S}}\left(\frac{\eta }{4}-c\right)}-\alpha c_{2}e^{-\frac{\eta }{2k_{T}}\left(\frac{\eta }{4}-c\right)}\right)}^{\prime \prime }=0.$$

The solution can be derived with quadrature and has the form of the following:

(17) $$\begin{array}{c} g\left(\eta \right)=e^{-\frac{\eta }{\nu }\left(\frac{\eta }{4}-c\right)}\left(c_{5}\cdot erf\left[\frac{1}{2}\sqrt{-\frac{1}{\nu }}\eta +\frac{c}{\sqrt{-\nu }}\right]+c_{6}-\right.\\ \frac{Gk_{T}k_{S}}{k_{S}k_{T}\left[2k_{S}-\nu \right]\left[2k_{T}-\nu \right]}\times \left[\beta c_{4}\left\{2k_{T}-\nu \right\}e^{\left\{\frac{1}{4\nu }-\frac{1}{8k_{S}}\right\}\eta^{2}+\left\{-\frac{c}{\nu }+\frac{c}{2k_{S}}\right\}\;\eta }-\right.\\ \left.\left.\alpha c_{2}\left\{2k_{S}-\nu \right\}e^{\left\{\frac{1}{4\nu }-\frac{1}{8k_{T}}\right\}\eta^{2}+\left\{-\frac{c}{\nu }+\frac{c}{2k_{T}}\right\}\;\eta }\right]\right). \end{array}$$

**Figure 3:**
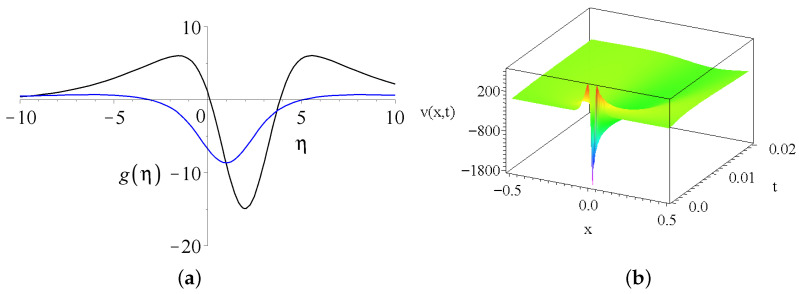
(**a**) Two shape functions of the velocity equation Equation (17). The common parameters of $\left(G,c_{2},c_{4},c_{6}\right)$ are (10,2,−6.2,1). The black and the blue curves have different values of the parameters $\left(k_{S},k_{T},\alpha ,\beta ,\nu ,c\right)$, namely (0.4,1.3,0.8,38.18,1) and (0.4,1.6,5.3,2.8,8.18,0.5), respectively. (**b**) The velocity distribution v(x,z=0,t) with the parameters of the black curve, respectively.

We analyze the real solutions only; therefore, we set $c_{5}=0$. It is easy to see that for $c_{4}=c_{2}$, α=β and $k_{T}=k_{S}$, the two exponential functions cancel each other because of the two opposing competing diffusion effects. The $k_{T},\;k_{S}$ and ν are still responsible for the FWHM of the peaks. It is also clear from the formula that for $\nu =2k_{s}=2k_{T}$ the velocity function becomes infinite. Large ν viscosity causes a small velocity because it stands in the denominator. Figure 3a shows us two different g(η) shape functions with different parameter sets. We can see different kinds of linear combinations of exponential-type functions (note that there is the product of a Gaussian and an exponential function in the formula). The results are now so exciting. Either we have a peak with a global maximum or minimum or two peaks with a minimum in between.

Figure 3b presents the velocity distribution $v\left(x,\;z=0,\;t\right)=t^{-\frac{1}{2}}\cdot g\left(xt^{-\frac{1}{2}}\right)$ for the parameters of the black curve. Note that the γ=1/2 exponent is responsible for the quick temporal decay.

**Figure 4:**
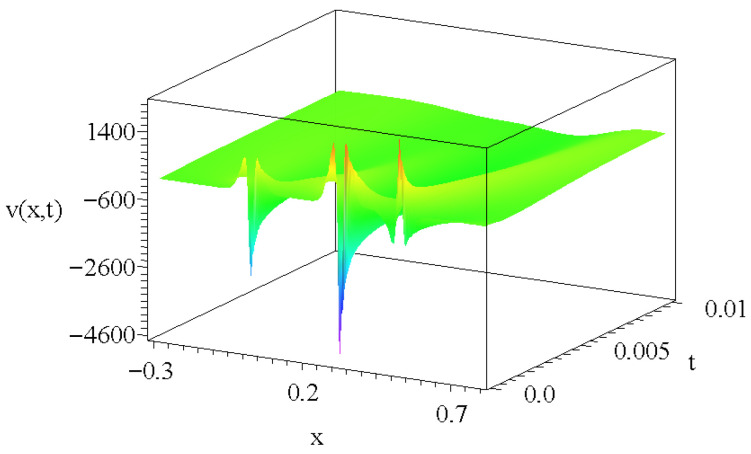
A possible velocity distribution as a linear combination of three real solutions in the form of $v\left(x,y=0,t\right)=\sum_{i=0}^{2}C_{i}t^{-\frac{1}{2}}\cdot g\left(\left[x-x_{i}\right]t^{-\frac{1}{2}}\right)$, where $x_{i}$ are (0,0.3,0.5). The physical parameters are the same as in Figure 3b). The linear combination parameters $C_{i}$s are (1,1.8,−0.9), respectively.

To emphasize the linearity of the velocity distribution Equation (16), Figure 4 presents a linear combination of three real solutions of Equation (17) for different $c_{i}$ integration constants and when the spatial coordinates are shifted. In principle, an arbitrarily large number of solutions could be summed up, resulting in very complex velocity distributions in double-diffusive convection systems.

In the following section we will study the irreversible nature of the double-diffusion process. The general theory how entropy production can be evaluated can be found in [27], based on the form of the temperature and density gradients. Entropy production is mostly derived in classical systems; however, it can be evaluated on quantum scales as well [60]. The entropy production due to the temperature inhomogeneities are as follows: $\sigma_{T}^{\left(irr\right)}$

(18) $$\sigma_{T}^{\left(irr\right)}=\lambda {\left(\frac{\nabla T}{T}\right)}^{2}.\;$$

where the λ is the heat conductivity of material, which may be expressed as $\lambda =\rho c_{p}k_{T}$, with ρ the density and $c_{p}$ the specific heat. Evaluating this form from Formula (14), one arrives at the following:

(19) $$\sigma_{T}^{\left(irr\right)}=\lambda \frac{1}{8}\frac{{\left(-x-y+2\sqrt{t}c\right)}^{2}}{t^{2}k_{T}^{2}}.$$

Next we evaluate the entropy production due to the inhomogeneity in concentration. Based on formula Equation (19) of chapter III of Ref. [27], the variation in chemical potential of component *l* contributes to the entropy production by a term

(20) $$\sigma_{S}^{\left(irr\right)}=-\frac{1}{T}j_{l}\left(T\nabla \left[\frac{\mu_{l}}{T}\right]\right).\;$$

In our case $j_{l}=j_{S}=-k_{S}\partial_{x}S$ is the current due to the salt concentration inhomogeneity. The $\mu_{l}$ is the chemical potential of salt—for relatively small concentrations. It may be considered $\mu_{l}=\mu_{S}≃RTln\left(S/S_{0}\right)$, where $S_{0}$ is a reference concentration. Applying the relation (20), for the entropy production related to the concentration inhomogeneity, we have

(21) $$\sigma_{S}^{\left(irr\right)}=k_{S}R\frac{{\left(\partial_{x}S\right)}^{2}}{S}.\;$$

Similarly to Equation (19) the final formula reads as follows:

(22) $$\sigma_{S}^{\left(irr\right)}=k_{S}R\frac{1}{8}\frac{{\left(-x-y+2\sqrt{t}c\right)}^{2}}{t^{2}k_{S}^{2}}.\;$$

Both formulas are quadratic in the spatial coordinate and therefore have inverse temporal dependencies.

### 2.2. The Role of Possible Additional Source Terms

We can see that our obtained self-similar solutions are far from being complicated and have no extra peculiarity. This is due to the fixed numerical values of all self-similar exponents. The Rayleigh-Bènard convection, which is also a fluid dynamical system with coupled heat conduction, has a much broader self-similar symmetry because one of the self-similar exponents remains free. Focusing in this direction to generalize the double-diffusive convection system, we may consider addition terms like a source in the temperature convection equation.

A straightforward way is taking a source term which is an arbitrary function of the temperature n(T); in this sense, Equation (3) is changed to the following:

(23) $$T_{t}+uT_{x}+vT_{z}-k_{T}\left(T_{xx}+T_{zz}\right)=n\left(T\right).\;$$

Keeping all exponents fixed to 1/2, it can be easily shown that the linear source terms should have the form of $n\left(T\right)=\frac{d\cdot T}{t}$. Here *d* is the strength of the source (if positive) or sink (when negative) and it fixes the proper physical dimension. The 1/t time-dependent factor is needed to have the proper temporal asymptotic. We tried additional power-law dependent source terms as well; only the square root $n\left(T\right)=\frac{d\cdot T^{1/2}}{t^{5/4}}$ has a trivial analytic solution of h(η)=0. (We can easily imagine a periodic driving term as well, but that should assume a traveling wave analysis which could be the topic of a possible forthcoming analysis. Such systems usually have Mathieu functions in their solutions.) These show interesting reconstructions of initial conditions with possible source in diffusion problems one may find in Ref. [61].

Considering the $\tilde{t}=t-t_{0}$ transformation, the singularity can be shifted, having smooth functions. The corresponding ODE for the temperature shape function is now slightly changed to the following:

(24) $$-\frac{h}{2}-\frac{\eta h^{\prime }}{2}+ch^{\prime }-2k_{T}h^{\prime \prime }=dh,$$

positive *d* values refer to the source. The solutions read as follows:

(25) $$h\left(\eta \right)=c_{1}M\left(\frac{1}{2}+d,\frac{1}{2},-\frac{{\left[2c-\eta \right]}^{2}}{8k_{T}}\right)+c_{2}U\left(\frac{1}{2}+d,\frac{1}{2},-\frac{{\left[2c-\eta \right]}^{2}}{8k_{T}}\right),$$

where M(,,) and U(,,) are the Kummer’s M and Kummer’s U functions with the usual integral constants of $c_{1}$ and $c_{2}$. For more information about Kummer’s functions see [59].

As a definition, consider the series expansion of M(,,)

(26) $$M\left(a,b,z\right)=1+\frac{az}{b}+\frac{{\left(a\right)}_{2}z^{2}}{{\left(b\right)}_{2}2!}+\ldots +\frac{{\left(a\right)}_{n}z^{n}}{{\left(b\right)}_{n}n!},$$

with the ${\left(a\right)}_{n}=a\left(a+1\right)\left(a+2\right)\ldots \left(a+n-1\right),{\left(a\right)}_{0}=1$ which is the so-called rising factorial or Pochhammer’s Symbol [59]. In our present case *b* has a fixed non-negative integer value, so none of the solutions have poles at b=−n. For the Kummer’s function *M*, when the parameter *a* has negative integer numerical values (a=−m), the solution is reduced to a polynomial of degree *m* for the variable *z*. In other cases a≠−m we get a convergent infinite series for all values of a,b and *z*. There is a connecting formula between the two Kummer’s functions; *U* is defined from *M* via

(27) $$U\left(a,b,z\right)=\frac{\pi }{\sin(\pi b)}\left(\frac{M[a,b,z]}{\Gamma [1+a-b]\Gamma [b]}-z^{1-b}\frac{M[1+a-b,2-b,z]}{\Gamma [a]\Gamma [2-b]}\right),$$

where Γ(a) is the Gamma function [59]. It is clear that the structure of the irregular Kummer’s U function is much more complicated.

**Figure 5:**
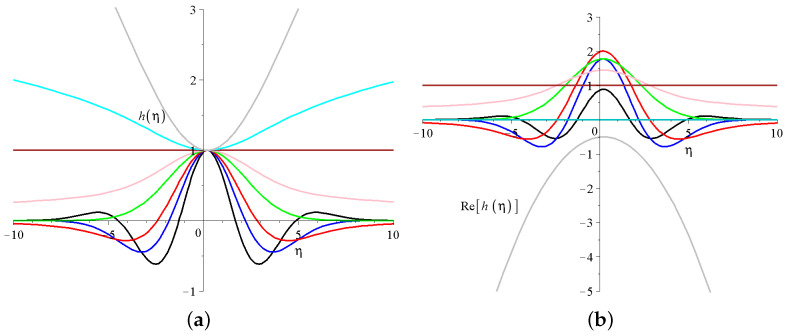
(**a**) The Kummer’s M part of the shape functions of the temperature Equation (25). The common parameters are $(c_{1}=1,c_{2}=0,c=0.1$ and $k_{T}=1)$. The black, blue, red, green, pink, brown, cyan and gray lines are for numerical values of the source strength parameter *d* of (2,1,1/2,0;−1/4,−1/2,−1,−3/2). (**b**) The real part of the Kummer’s U part with the same parameters.

These very nice mathematical formulas do not help us much to visualize and to imagine how these functions look like for different parameters, especially for quadratic arguments. Therefore we present them for some parameter values. Figure 5a presents the regular Kummer’s M function parts of the solution Equation (25) for different *d* source values. The *c* former integral constants are just shifted parallel to the x axis, and the $k_{T}$ parameter defines the widths of the solutions. All these functions are real and regular in the origin.

It is important to emphasize that there are four parameter ranges that exist where the derived solutions behave qualitatively different:

- d<−1/2, where the derived solutions are divergent for large ηs; these are the cyan and the gray lines on Figure 3a. If the first parameter of the Kummer’s M function is a negative integer then the function is a finite order polynomial in η. A nice example is $d=-\frac{3}{2}$ where
  (28) $$M\left(-1,\frac{1}{2},-\frac{{\left(2c-\eta \right)}^{2}}{8k_{T}}\right)=M\left(-1,\frac{1}{2};-\frac{c^{2}}{2k_{T}}\right)-\frac{c}{k_{T}}\eta +\frac{1}{4k_{T}}\eta^{2}.$$
  Note that the first term on the right hand side is a constant (formally Kummer’s function of the first kind M(,,) is equivalent to the generalized confluent hypergeometric series with the notation of F11(,,)).
  The smaller the first negative parameter of the Kummer’s function, the larger the power of the polynomial. Thanks to the δ=1/2 exponent, the final T(x,z,t) temperature distribution will be decaying, but we will see that this parameter regime will not attract the largest interest among the solutions.
- d=−1/2, the solution is constant on the whole η axis, this is presented by the brown line.
−1/2≤d≤0, the solution is positive on the whole axis, and has a decay to zero at large ηs. Such solutions are plotted with pink and green lines. These are well-behaving solutions with a global maxima in the origin, and in this sense similar to Gaussian solutions.
- 1/2<d, the solutions has a maxima in the origin following quick oscillatory decay to zero with growing number of zero transitions as d growing. Black, blue and red curves present such solutions. Unfortunately, the defining series of the Kummer’s M function Equation (26) converges very slowly for highly oscillatory functions.
  In some sense these are the most interesting solutions.

**Figure 6:**
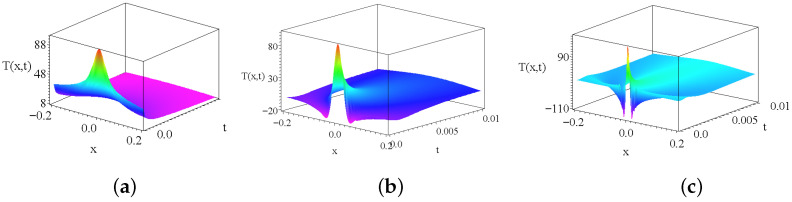
The Kummer’s M part of the temperature distribution. The left subfigure (**a**) is for d=−1/3, the middle subfigure (**b**) is for D=1/3 and the right subfigure (**c**) is for the parameter d=2, respectively. The additional common parameters are the same in all cases $\left(k_{T}=c=1\right)$, respectively.

For completeness we show on Figure 5b how the irregular Kummer’s U solutions behave. It can be shown that the Kummer’s U functions with quadratic arguments are finite polynomials, and the first arguments are negative integer or half integer values. We present only the real part of the solutions. Note that the general properties are very similar. There are oscillatory and decaying solutions. There are divergent solutions and there is a finite constant and a constantly zero solution as well. To have a complete overview, Figure 6 shows three temperature distributions given with the Kummer’s M functions. We considered physically relevant decaying solutions in three different parameter regimes.

We gained considerable experience with the Kummer’s U and Kummer’s M functions [17,18,19,20] in recent years. Usually, an additional Gaussian weight function was involved in the solutions, but the general properties were very similar. The main difference to our former solution is that a physical parameter (strength of the heat source) works as an index of the solution and additionally we always have a $t^{-1/2}$ prefactor in the final dynamical variable which automatically gives us a temporal decay at infinite times.

According to Equation (18) the entropy production can be easily derived, giving us a cumbersome but finite formula of

(29) $$\sigma_{T}^{\left(irr\right)}=8\lambda \frac{{\left[\left(d-\frac{{\left[2c-\frac{x+y}{t^{1/2}}\right]}^{2}}{8k_{T}}\right)M\left(\frac{1}{2}+d,\frac{1}{2},-\frac{{\left[2c-\frac{x+y}{t^{1/2}}\right]}^{2}}{8k_{T}}\right)-d\cdot M\left(-\frac{1}{2}+d,\frac{1}{2},-\frac{{\left[2c-\frac{x+y}{t^{1/2}}\right]}^{2}}{8k_{T}}\right)\right]}^{2}}{{\left[{\left(2c-\frac{x+y}{t^{1/2}}\right)}^{2}\cdot t\cdot M\left(\frac{1}{2}+d,\frac{1}{2},-\frac{{\left[2c-\frac{x+y}{t^{1/2}}\right]}^{2}}{8k_{T}}\right)\right]}^{2}}.$$

Note the 1/t temporal decay.

To complete our investigations we have to analyze the behavior of the velocity field. Considering only real solutions, we take the Gaussian solution for the salinity and the Kummer’s M functions for the heat distribution; we can formulate the final ODE as follows:

(30) $$\begin{array}{c} -\frac{g}{2}-\frac{\eta g^{\prime }}{2}+cg^{\prime }-2\nu g^{\prime \prime }+G\left[\beta c_{4}{\left(e^{-\frac{\eta }{2k_{S}}\left(\frac{\eta }{4}-c\right)}\right)}^{\prime \prime }-\right.\\ \left.\alpha c_{1}M{\left(\frac{1}{2}+d,\frac{1}{2},-\frac{{\left[2c-\eta \right]}^{2}}{8k_{T}}\right)}^{\prime \prime }-\alpha c_{2}U{\left(\frac{1}{2}+d,\frac{1}{2},-\frac{{\left[2c-\eta \right]}^{2}}{8k_{T}}\right)}^{\prime \prime }\right]=0, \end{array}$$

for better transparency, we marked and did not complete the second derivatives in the equation. Unfortunately, we could not find the closed form for the solutions for the general Kummer’s functions. However, if the power of the series of the Kummer’s function is not larger than four, then the ODE has an exact solution:

(31) $$\begin{array}{c} -\frac{g}{2}-\frac{\eta g^{\prime }}{2}+cg^{\prime }-2\nu g^{\prime \prime }+G\left[\beta c_{4}{\left(e^{-\frac{\eta }{2k_{S}}\left(\frac{\eta }{4}-c\right)}\right)}^{\prime \prime }-\right.\\ \left.\alpha c_{1}{\left(a_{0}+a_{1}\eta +a_{2}\eta^{2}+a_{3}\eta^{3}+a_{4}\eta^{4}\right)}^{\prime \prime }\right]=0, \end{array}$$

where the $a_{i}$ coefficients depend on the parameters of $c,k_{T}$ in a complicated way. The solution is exhaustively long and contains numerous Gaussian and error function terms. The direct form is given in the Appendix A at the end of the study.

## 3. Summary and Outlook

We investigated the double-diffusion convection flow system, which means that two competing diffusion processes are coupled to the momentum equation. Examples of real processes are heat and salt convection in water. Our self-similar Ansatz easily gave the Gaussian and error functions for the salinity, temperature and flow velocity distributions, which are less than our former expectations. The reason is that all self-similar exponents had a given $+\frac{1}{2}$ numerical value. Additionally, the entropy production—which is a measure of irreversibility—was derived as well. The derived function has a $1/t^{\alpha }$ asymptotic behavior for large times and fixed positions. To deepen our analysis we considered an additional cooler or heater source term in the temperature convection equation which drastically opened the horizon of possible solutions. The heat distribution function becomes the Kummer’s M or Kummer’s U function. The strength of the heat source becomes the first parameter of the temperature distribution, which is a well-understood mathematical feature. Unfortunately, the final velocity distribution cannot be evaluated analytically for all temperature distribution parameters; however, it was possible up to a considerable order of expansion of the function of the temperature. For future research, we may consider non-linear heat conduction mechanisms and non-linear (beyond Boussinesq’s approximation) salinity diffusivity. 

## Data Availability

All used data are given in the manuscript.

## Appendix A

As a direct example, consider d=−5/2

(A1) $$\begin{array}{cc} & M\left(-2,\frac{1}{2},-\frac{{\left(2c-\eta \right)}^{2}}{8k_{T}}\right)=M\left(-2,\frac{1}{2};-\frac{c^{2}}{2k_{T}}\right)-2cM\left(-1,\frac{3}{2};-\frac{c^{2}}{2k_{T}}\right)\eta +\\ & \frac{2c^{2}+3M\left(-1,\frac{3}{2},-\frac{c^{2}}{2k_{T}}\right)k_{T}}{6k_{T}^{2}}\eta^{2}-\frac{c}{6k_{T}^{2}}\eta^{3}+\frac{1}{48k_{T}^{2}}\eta^{4}, \end{array}$$

After performing the double differentiation, we get the following:

(A2) $$i{\left(\eta \right)}^{\prime \prime }=\frac{2+3M\left(-1,\frac{3}{2},-\frac{1}{2k_{T}}\right)}{3k_{T}^{2}}-\frac{\eta }{k_{T}^{2}}+\frac{\eta^{2}}{4k_{T}^{2}}.$$

We name the first constant term of the right hand side with ‘A’. After substituting this formula to Equation (31) the solution can be derived with quadrature thanks to Maple 12. Additional exhausting simplification by hand gives us the following:

(A3) $$\begin{array}{cc} & g\left(\eta \right)=\frac{e^{-\frac{{\left(\eta -2\right)}^{2}}{4\nu }}erf\left(\frac{\eta -2}{2\sqrt{-\nu }}\right)}{3k_{T}^{2}\sqrt{-\nu }}\cdot \left(-3C_{1}\sqrt{-\nu }k_{T}^{2}e^{\frac{1}{\nu }}+2G\alpha A\sqrt{\pi }k_{T}^{2}-4G\alpha \sqrt{\pi }\right)+\\ & C_{2}e^{-\frac{\eta^{2}}{4\nu }}\cdot e^{\frac{\eta }{\nu }}-\frac{G}{6k_{T}^{2}\left(2k_{S}-\nu \right)}\cdot \left(c_{4}\beta k_{T}^{2}e^{-\frac{\eta (\eta -4)}{8k_{S}}}-4\nu \alpha Ak_{T}^{2}+8k_{S}\alpha Ak_{T}^{2}-\right.\\ & \left.4\nu \alpha \eta +8\nu \alpha -16k_{S}\alpha -\nu \alpha \eta^{2}+2k_{S}\alpha \eta^{2}+4\nu \alpha -k_{S}\nu \alpha \right). \end{array}$$

Note that the solution contains Gaussian, exponential, η and $\eta^{2}$ type dependencies only. The complexity comes from the large number of parameters.
